# Mechanical Properties of Optical Fiber Strain Sensing Cables under *γ*-Ray Irradiation and Large Strain Influence

**DOI:** 10.3390/s20030696

**Published:** 2020-01-27

**Authors:** Arianna Piccolo, Sylvie Delepine-Lesoille, Etienne Friedrich, Shasime Aziri, Yann Lecieux, Dominique Leduc

**Affiliations:** 1 French National Radioactive Waste Management Agency (Andra), 92298 Chatenay-Malabry, France; 2 Laboratoire GeM UMR 6183, Université de Nantes, 44000 Nantes, France; 3 Solifos AG, Fiber Optic Systems Klosterzelgstrasse 41, CH-5210 Windisch, Switzerland

**Keywords:** distributed optical fiber strain sensing cable, Brillouin scattering, Rayleigh scattering, strain sensing cable characterization, elasto-plastic behavior, strain sensitivity coefficients

## Abstract

Optical fiber strain sensing cables are widely used in structural health monitoring; however, the impact of a harsh environment on them is not assessed despite the huge importance of the stable performances of the monitoring systems. This paper analyzes (i) the impact of the different constituent layers on the behavior of a strain sensing cable whose constitutive materials are metal and polyamide, (ii) the radiation influence on the optical fiber strain sensing cable response (500 kGy of γ-rays), and (iii) the behavior of the cable under high axial strain (up to 1%, 10,000 με). Radiation impact on strain sensitivity is negligible for practical application, i.e., the coefficient changes by 4% at the max. The influence of the composition of the cable is also assessed: the sensitivity differences remain under 15%, a standard variation range when different cable compositions and structures are considered. The elasto-plastic behavior is at the end evaluated, highlighting the residual strain (about 1600 με after imposing 10,000 με) of the cable (especially for metallic parts).

## 1. Introduction

Structural health monitoring (SHM) is an important topic in society currently, as buildings’ maintenance must be the most cost and time effective as possible. Distributed optical fiber sensing, to monitor strain and other parameters, is found to be a useful tool thanks to optical fiber’s desirable features (versatility, dimensions, measurement range, insensitivity to electromagnetic fields, etc.) [1,2]. Distributed optical fiber sensors are sensitive to strain and temperature thanks to the backscattering mechanisms occurring inside the fiber, namely Brillouin and Rayleigh scatterings. These mechanisms allow us to use optical fibers as distributed sensors, reaching many sensing points in the distance with only one sensor. The light that propagates inside the core of the fiber is partially backscattered to its origin, carrying back information on the thermal and mechanical state of the fiber. Once the backscattered light frequency is recollected, it is possible to obtain the frequency shift Δν at every point along the fiber between the injected and backscattered light, which depends on the fiber conditions as:

(1) $$\Delta \nu =C_{T}\Delta T+C_{\varepsilon }\Delta \varepsilon ,$$

where $C_{T}$ and $C_{\varepsilon }$ are the thermal and strain sensitivity coefficients of the considered sensor. Optical fibers’ sensitivity coefficients are in the order of $C_{T}^{B}=1$ MHz/°C for temperature and $C_{\varepsilon }^{B}\;=\;0.05$ MHz/με for strain sensitivity for Brillouin scattering [3], while for Rayleigh scattering, the coefficients are respectively in the order of $C_{T}^{R}=-1.5$ GHz/°C and $C_{\varepsilon }^{R}=-0.15$ GHz/με [4].

In SHM applications, optical fibers are often inserted into cables to improve the sensitivity performances, enhance their mechanical robustness, and protect the fiber from the harsh environment in which it is employed. The structure of the cable may however have an influence in both the short and the long term sensing characteristics of the fiber. A harsh environment, in fact, can affect not only measurement results, but also the mechanical behavior of the sensor itself and its durability. In the majority of the applications, however, the maintenance over the monitoring period is possible only on the interrogation instrument, while the sensor is definitively embedded in its environment and cannot be accessed to be repaired nor replaced. It is therefore important to select not only the best interrogation method, to reduce at maximum the measurement errors, but the sensing cable as well, able to resist the application’s load level and harsh environment over the needed monitoring period. In nuclear structures’ monitoring, such as nuclear power plants’ operation and dismantlement phases, physics reactors (CERN, etc.), or the space industry, sensing systems face radiation while the monitoring period must exceed 50 years. For the French project of the deep geological disposal facility for high level and intermediate level long lived radioactive waste (known as Cigéo), the application includes the presence of radiation, high temperatures (up to 90 °C), and chemicals (H_2_). Investigations on the impact of these harsh conditions on optical fibers in their primary coating have been and are still being carried out, in order to select the best optical fiber composition and interrogation method [5,6]. Nevertheless, as distributed optical fiber sensors (DOFS) are often put into a cable when employed on-field, their composition and the structure of the cable must be carefully selected to be sufficiently resistant and, at the same time, keep as much as possible the elasticity of the sensor. The external sheath should also be chosen in order to maintain or even enhance its sensitivity to the measured variable. For this reason, tests should be carried out in order to analyze the physical and sensitivity characteristics of pre-existent or brand new optical fibers and optical fiber cables. Much work has already been devoted to optical fibers (in primary coating): for example, in [7], the mechanical properties and strain transferring mechanism of optical fiber sensors were analyzed, on the different layers of a fiber Bragg grating (FBG) , while in [8], the role of the coating was studied for strain transfer. In [9], the concept of thermal stability in optical fibers was clarified, and in [10], the coating thermal stability and mechanical strength at elevated temperatures of optical fibers were evaluated on different samples. The physical properties of the coating were also evaluated in [11], where the elasto-plastic bond mechanics of the fiber coating were evaluated, while in [12], a fatigue test was carried out assessing the performance stability of DOFS over two million load cycles.

Regarding cables, however, the bibliography is not so wide. As strain sensing cables are deployed to measure the strain of the structure, many papers are focused on the analysis of the strain transfer function, i.e., to know how much of the structure’s strain is transferred to the fiber: $\varepsilon_{\mathrm{FO}}\left(s\right)=\varepsilon_{\mathrm{struct}}\left(s\right)⊗\mathrm{MTF}\left(s\right)$. The mechanical transfer function MTF(s), which translates the strain of the structure $\varepsilon_{\mathrm{struct}}\left(s\right)$ in the sensor strain $\varepsilon_{\mathrm{FO}}\left(s\right)$, represents the behavior of the sensor without the need to specify its physical and mechanical characteristics. The strain transfer function of different kinds of cables is already assessed (for example, [13,14]); however, the physical and sensitivity characteristics of strain sensing cables, as the elasto-plastic behavior and the impact of the protection layers on the optical fiber measurements, are not well considered in the literature. One exception is [15], where the strain sensitivity of different strain sensing cables was analyzed, with attention to the initial residual hysteresis.

The French national agency for radioactive waste management (Andra), for its monitoring needs, has selected some strain sensing cables, of which one has been already used for convergence monitoring tests [16]. This cable was then analyzed in order to understand its physical behavior under traction, as well as its mechanical characteristics and sensitivity. Another important aspect is the durability of the sensors in a harsh environment. The impact of radiation, alone or in combination with other environmental conditions (as high temperature), is well assessed for optical fibers [5,6,17,18]; however, it is not known for commercial strain sensing cables. For this reason, part of the tested strain sensing cable samples has also been irradiated, in order to check the radiation impact on the cable’s different layers and mechanical behavior.

In this paper, the mechanical properties and in particular elasticity characteristics of the same optical fiber strain sensing cable are analyzed under diverse conditions. The sensor was tested in its whole, with part of the protection removed and without other protection than the primary coating (i.e., bare fiber). Moreover, the cable’s characteristics are also evaluated under irradiation in order to determine the radiation impact on the behavior of the sensor. Lastly, a distinguishing aspect of this study is testing the response of an optical cable subjected to large strain. As any other sensor, an optical fiber cable is designed to operate in the elastic domain, i.e., for strain levels often less than 0.2% (2000 με). However, in the long run or due to unforeseen loads, this threshold may be exceeded. It is important then to understand if the acquired measures are reliable; to provide an answer, the optical fiber cables are tested up to 1% in strain (10,000 με), which is beyond what a sensor is expected to undergo on-site. After reporting the specimen’s characteristics (comprised of a summary of the sensors’ sensitivities) and the test procedure, the elasto-plastic behavior of the selected optical fiber strain sensing cable is shown.

## 2. Cable Mechanical Characterization: Motivation

In Cigéo, two kinds of long lived radioactive wastes, intermediate level (IL-LL) and high level (HL), will be hosted inside cylindrical repository cells with concrete and steel liners, respectively. These cells are located 500 m deep, inside a 120 m thick Callovo-Oxfordian claystone layer. For the French law, the repository must be reversible for the first one hundred years at least; therefore, a monitoring program will be implemented from the construction phase and throughout its operating life, to keep track of repository safety related parameters. What is more, it will also contribute to ensure the safety of the waste and the surroundings. The particular application however reduces the choice of the possible sensing systems, mostly due to the environmental conditions: the sensor must endure radiation up to 1 MGy (total dose received at the external surface of the metallic liner of “HL0” repository cells after a century of monitoring), maximum temperature around 90 °C, hydrogen presence, cells’ convergence (i.e., reduction of the cell’s vertical diameter) of 10 mm, and an orthoradial strain level (compression and traction) of about ±0.3% around the cell’s circumference (∼2700 με). Moreover, it has to be embedded in or fixed on the liner in order to avoid (i) most of the harsh environment’s impact and (ii) limiting space dedicated to the waste and handling spaces.

**Figure 1:**
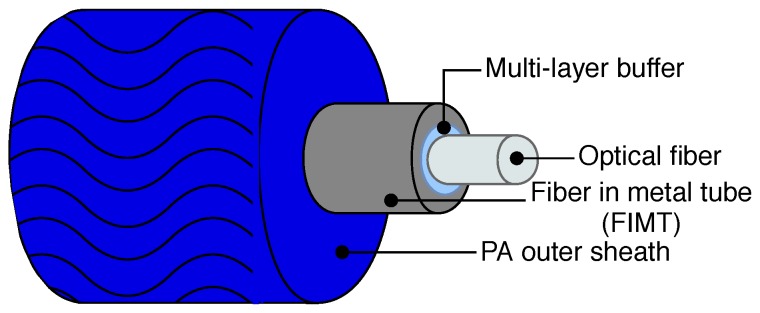
Schematic of the V9 type strain sensing cable, composed by a polyamide sheath of 3.2 mm in diameter, a steel tube (FIMT) of ∼0.9 mm in diameter, a multi-layer buffer, which helps the strain transfer, and a single mode fiber (SMF) (250 μm).

Following (1), temperature impacts optical fiber by simply shifting the frequency. Radiation effects on light propagation (frequency shift and attenuation) are attenuated if the fiber is fluorine doped [18], while it is known that a carbon coating helps the fiber to be hermetic to hydrogen absorption, which would otherwise lead to additional losses [19]. In order to limit the risk of the breaking of the sensor, which could not be replaced, the fiber should be protected in a cable to better endure the compression of the surrounding rock and last over time. For this reason, cables with a metallic structure are preferred as it is more durable than plastic, while a rough surface might help the strain transfer of the cable in concrete thanks to a higher level of bonding between the two materials. In the framework of the Cigéo project, to reduce cost and save time, it is preferred to focus on commercial products, when possible. If the market does not offer performances that fulfill the requirements, it is necessary to start specific developments. For this reason, Andra investigated many suppliers and analyzed different cables from over seven companies, and a good tradeoff between tensile strength and minimum curvature radius was found to be given by the BRUsens V9 type from Solifos AG. The V9 type is a 3.2 mm mini armored fiber optic strain sensing cable with an ∼0.9 mm central metal tube (FIMT, fiber in metal tube), a structured polyamide (PA) outer sheath, and one optical single mode fiber (SMF) inside. The design of the V9 type cable is depicted in Figure 1.

This cable was already tested by Andra in a surface test [16], revealing the suitability for convergence and strain monitoring. The same cable, which normally has a standard single mode fiber with higher curvature resistance inside, was used to develop a custom cable for Andra’s application. The resulting cable was the outcome of the insertion of a custom SMF, carbon coated for hydrogen hermeticity, fluorine doped for radiation hardening, in a commercial strain sensing cable (in this case, the V9 type). This custom SMF had a 0.3 wt% F doped core and a 2.3 wt% F doped cladding, a numerical aperture of 0.14, and a core diameter of 7.4 μm, with an attenuation at 1550 nm of 0.40 dB/km and an effective refractive index of about 1.439. Before using this cable on site, however, it is necessary to know its characteristics in sensitivity and durability. Once the fiber is protected in a cable, its characteristics may change as the composition (materials, dimensions, etc.) of the sensor changes. For these reasons, the tests presented in this paper were meant to assess (i) the sensitivity of the newly developed sensor, (ii) the influence of the different protective layers on the behavior of the sensor, and (iii) the impact of a harsh environment (radiation, in this case) on its performances. Radiation impacts both optical fibers’ attenuation and frequency shift. The radiation induced attenuation (RIA) and SNR issues were already thoroughly evaluated in [5] on optical fibers in their primary coating. As attenuation poorly depends on the presence of the cable and the total absorbed dose here considered was lower, those results could be seen as a worst case scenario and were therefore supposed to remain valid in this case. For this reason, the radiation impact on optical fiber sensing cables was focused only on the induced frequency shift.

## 3. Materials

**Figure 2:**
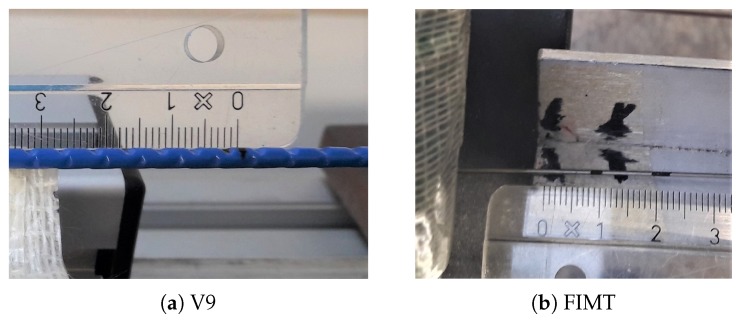
Tested cables: V9 (**a**) and FIMT (**b**) types.

In order to characterize the sensor, the tests were conducted not only on custom V9 type cable samples (Figure 2a), but also on their constitutive parts: the FIMT (with the custom radiation hard fiber inside; Figure 2b) and the naked fiber itself (only primary coating). Besides, the same analysis was carried out on standard commercial samples of the same types (V9, FIMT and bare fiber), which had an SMF G657 with acrylate coating. It is in fact interesting to assess whether the different fibers inside the cable influence in different ways the performances of the sensors. Furthermore, as the goal was also to assess the impact of radiation, part of the V9 and FIMT samples were previously irradiated up to 500 kGy, which is half of the absorbed total dose during the first 100 years of monitoring. The dose was the result of an irradiation campaign where bare optical fibers were irradiated up to 1 MGy [5]. The different distances of the cables from the irradiation source and their support in metal allowed them absorb less dose, i.e., up to 500 kGy. The dose rate was about 1.5 kGy/h. For the sake of comprehension, the different samples under tests are synthesized in Table 1 and will be so addressed from now on.

## 4. Methods

The samples were tested under traction cycles: the frequency shifts of the samples were acquired, using the Neubrescope NBX-7020 from Neubrex Co., Ltd., which is able to exploit Brillouin and Rayleigh scatterings with the pulse pre-pump Brillouin optical time domain analysis (PPP-BOTDA) and the tunable wavelength coherent optical time domain reflectometry (TW-COTDR), respectively, with the highest spatial resolution of 2 cm.

**Figure 3:**
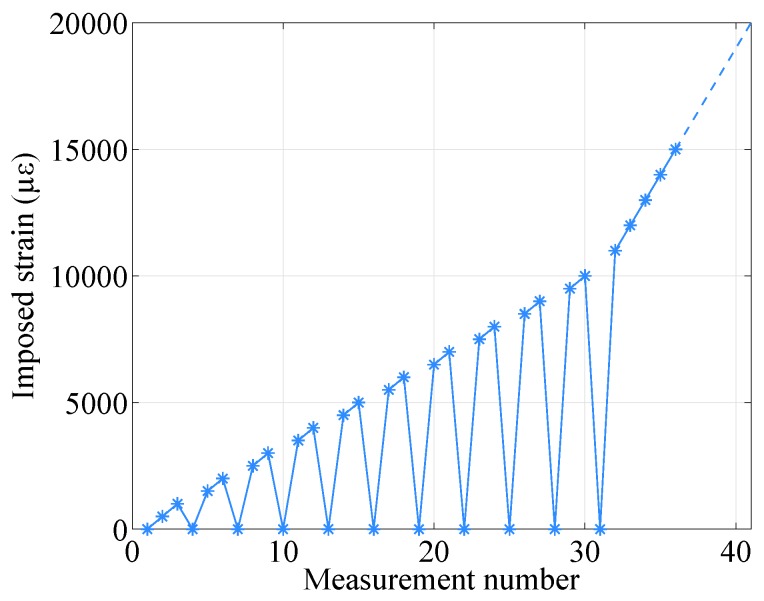
Traction cycle.

Traction tests were performed by fixing the samples on a 10 m manual traction bench. The samples were elongated using a winch, checking the new length with a ruler and a laser distance meter (millimetric precision). Measurements were acquired every 500 με (nominal value, 5 mm of elongation), while each 1000 με step, the sample was taken back to the initial position (no elongation) in order to check whether there was (or not) residual strain (i.e., to analyze the plastic strain of the sample). Another measurement was then acquired. This was performed up to 10,000 με, while for the FIMT type samples, the measurements back to zero were performed up to 7000 με. Once the maximum strain range was reached (1% of strain by the datasheet, i.e., 10,000 με), measurements were acquired every 1000 με (10 mm in elongation), without taking the sample back to its original position (no elongation), up to 30,000 με or up to the breaking point. The generalized traction cycle is depicted in Figure 3.

This kind of measurement cycle was conceived of to reach various goals: (i) practically measure the impact of elongation on the materials of the samples (i.e., the elasto-plastic behavior) and (ii) obtain the most representative sensitivity coefficient of the sample after pre-straining it, avoiding hysteresis (as explained in [15]). Moreover, the maximum strain expected in the Cigéo application is ±2700 με, which would be progressively reached over a period of 100 years in a monotonous fashion, in the reference scenario where radioactive waste packages are inserted right after the construction of the cells. However, as the implementation scenario is not already settled, it could be possible that the growth in strain and convergence is not fully monotonic. For example, the strain is non-monotonic if waste packages are inserted long after the construction, especially for concrete liners (IL-LL waste repository cells). The insertion of the packages would cause an immediate change in the strain of the structure, which would be already influenced by creep and shrinkage. Along with this, the concrete would also be affected by seasonal thermal cycles, taking to an even more non-monotonous behavior. Nevertheless, after the insertion of the waste packages, there would be a rise in temperature, causing a dilatation of the structure (especially the steel liner of HL waste repository cells). For all these reasons and in order to anticipate extraordinary situations, as well as to extend the study beyond the nominal conditions, this research explored the effect on the sensors of both “non-monotonous” strain and of very large strains, up to 10,000 με.

During elongation, the cabled samples, especially of the V9 type, slipped from the anchoring due to difficulties in the fixation of samples with a diameter greater than a millimeter. This led to an error (between the desired strain value and the one obtained in reality after the slippage) that remained however under 7%–8%, and it grew in a distributed and homogeneous way from 0 με to the maximum elongation reached by the sample. In this way, the correct analysis of the samples was guaranteed.

Measurements were taken with a resolution of 20 cm and a sampling of 10 cm, obtaining about 100 measurement points over the 10 m bench (excluding connection cables). It has also to be specified that, as all tests were performed in the same period of time and in the same place, the temperature was supposed to be stable (differences in the order of ±2 °C). Therefore, the measured frequency shift was attributed solely to the imposed traction. The results presented in the following are the outcome of the analysis of one sample of each specimen summarized in Table 1. In this case, only measurements up to 10,000 με were considered.

## 5. Strain Sensitivity Coefficients

**Figure 4:**
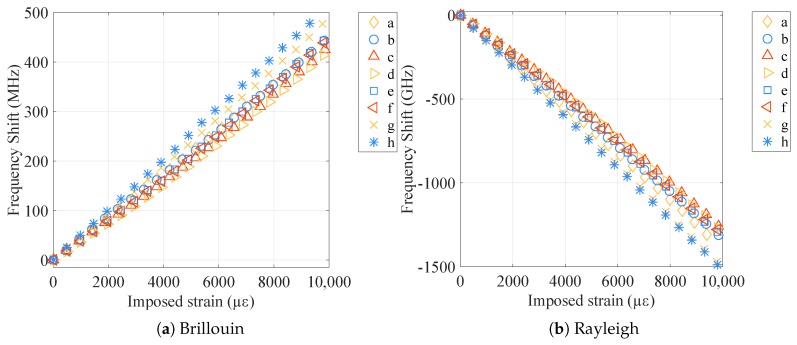
Frequency shift over strain curves for all the tested samples, for Brillouin and Rayleigh scatterings.

The strain sensitivities of the tested samples were obtained by averaging the frequency shifts Δν, obtained by interrogating the samples with Brillouin and Rayleigh scatterings, over the central 9 m of the samples (to avoid measurements on the fixations) and dividing them by the imposed strain range. The curves Δν over strain so obtained for Brillouin and Rayleigh scatterings are plotted in Figure 4. The strain sensitivities of both types of bare fibers were the ones that were closer to the standard values, $C_{\varepsilon }^{B}=0.050$ MHz/με for Brillouin and $C_{\varepsilon }^{R}=-0.15$ GHz/με for Rayleigh, while for the cables, the coefficients stayed respectively around $C_{\varepsilon }^{B}=0.045$ MHz/με and $C_{\varepsilon }^{R}=-0.13$ GHz/με.

**Figure 5:**
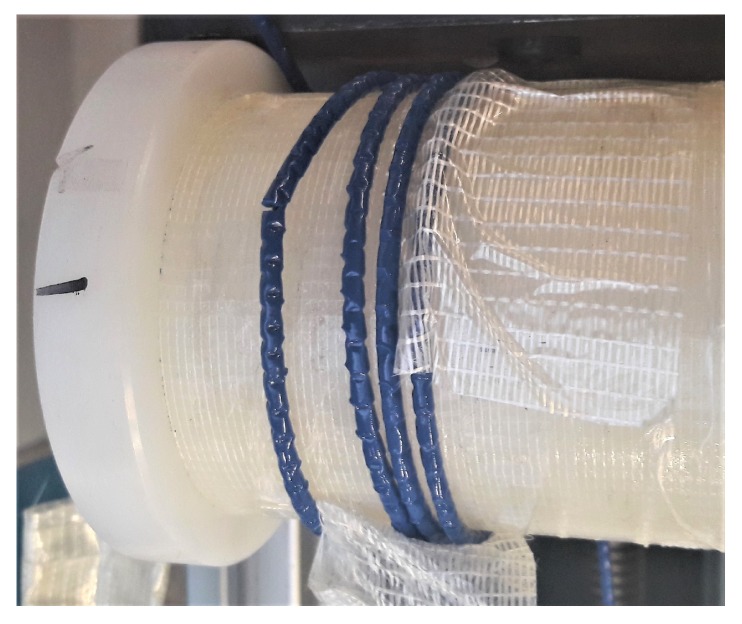
Impact of the radiation on the cable: radiation reduces the elasticity of the plastic, causing cracks when curved.

Observing the results, especially the cabled samples (V9 and FIMT types), it is noticeable how the strain sensitivities do not differ very much from one sample to another. The strain sensitivity differences between the final sensor (V9 type) and the original bare fiber went from 9% (standard type) to 12% (custom radiation hard), revealing the possibility to insert the desired fiber into the cable, keeping the information on its strain sensitivity as much as possible. The sensitivity differences remains thus under 15%, a standard variation range when different cable compositions and structures are considered. When it comes to analyzing the impact of radiation on the sensor, the sensitivity difference between irradiated and not irradiated samples was as low as 1% (FIMT type) and 4% (V9 type), which means that the sensitivity remained stable. This is a very promising result: if we suppose radiation influence as linear, the 4% in error over 500 kGy would mean an error in strain of 8% in 100 years (1 MGy), i.e., only about 220 με over 2700 με. Moreover, for the F doped fiber, the radiation influence is not linear; it tends to saturate in a parabolic way [5], which means that the error in reality would be even less. In every case, sensitivity coefficient values for irradiated samples were lower than for those not irradiated. Most of the radiation impact was exerted on the physical elasticity of the cable: the PA outer sheath became more rigid and less elastic due to radiation [20], leading to more cracks during handling and elongation and, therefore, to break sooner than non-irradiated samples (Figure 5).

With the exception of the fibers, the standard samples, and the not irradiated V9 type, some cables broke during the test. The custom irradiated V9 type broke, reaching the nominal value of 12,000 με, while custom FIMT type cables broke at 21,000 με and 29,000 με, respectively, for the pristine and the irradiated one. In practice, the only cable that suffered from radiation influence was the V9 type due to the impact on the polyamide, while the FIMT type cables broke mainly due to their structure. In all cases, if breaks occurred during traction, it was always after the 10,000 με; therefore, the datasheet guaranteed strain range was maintained. These results are very promising for the use of such a cable in an application where radiation is present.

## 6. Elasto-Plastic Behavior

**Figure 6:**
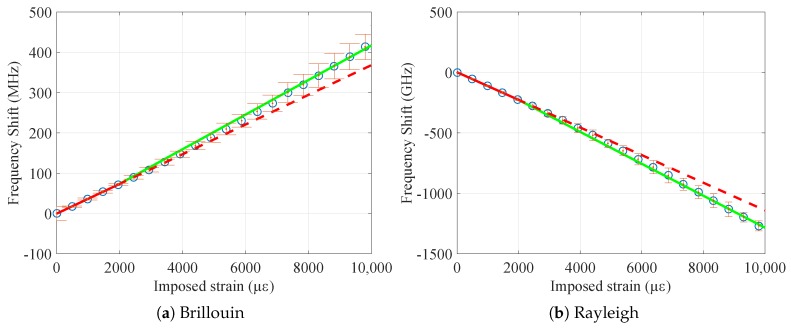
Detail of the mechanical behavior of the FIMT standard type sample (Sample d). The curve presents two zones with different slopes (highlighted in red and green): this represents the plasticity of the steel of which the FIMT is made.

As the cable is partially composed of steel, which is the most rigid component of the cable and tends to show a plastic behavior after a certain strain (typically, 0.2% [21], 2000 με), it was interesting to look for the possible plastic behavior of the tested cabled samples (FIMT and V9 types). Let us take as an example the standard FIMT (sample *d* in Table 1), in order to directly observe the behavior of the steel protecting the fiber. In Figure 6a,b, the frequency shift over the strain curve is plotted, respectively for Brillouin and Rayleigh scatterings.

The uncertainty of the measurements is reported as error bars, while the slope of the curve is calculated separating the strain behavior into two zones: before and after 2000 με. It is visible how the linear regressions of these two zones are different: before 2000 με (red line), the strain coefficient (i.e., the slope) is smaller than afterwards (green line), showing a possible plastic behavior of the sample due to traction. This is valid also for the other samples, the strain coefficient values of which are reported in Table 2.

**Figure 7:**
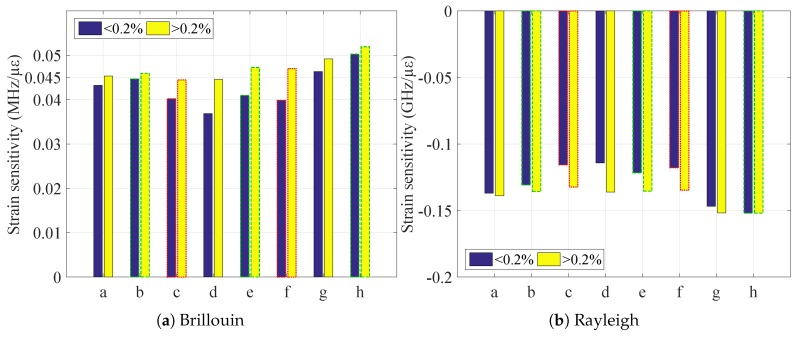
Strain sensitivity coefficients for different linear fittings: “<0.2%” and “>0.2%” represent the fit done considering only the values before or after 2000 με. The different contours of the bars define the type of sample: the black and straight line are the standard samples, the green and dashed the custom not irradiated, and the red and dotted the custom irradiated.

To make it more immediate to evaluate, the strain sensitivities are plotted in Figure 7.

The biggest difference between the sensitivities of the two identified zones (and therefore, the biggest plastic effect) appears to be exerted on the FIMT, as the V9 type is composed also of the external PA layer, which limited the plastic behavior of the steel.

**Figure 8:**
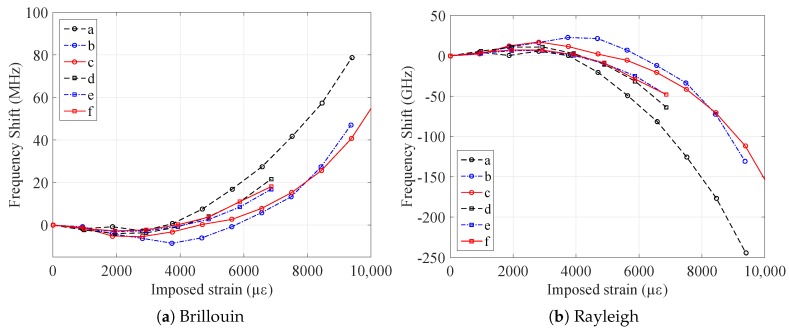
Residual frequency shift of the samples after elongation (represented in the *x* axis) of the cabled samples (V9 and FIMT) for the three tested types of condition (standard fiber, custom radiation hard fiber, and custom radiation hard fiber irradiated).

This behavior should be then confirmed looking at the measurements performed when the samples were at their original position, i.e., when they were not elongated. The frequency shifts of the samples, obtained by interrogating the samples via Brillouin and Rayleigh scatterings at their original position, are plotted in Figure 8a and Figure 8b respectively.

**Figure 9:**
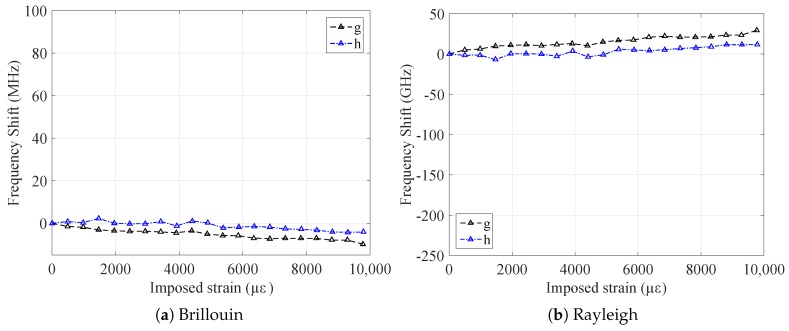
Residual frequency shift of the samples after elongation (represented in the *x* axis) of the fiber samples for the two tested types of conditions (standard fiber, custom radiation hard fiber).

**Figure 10:**
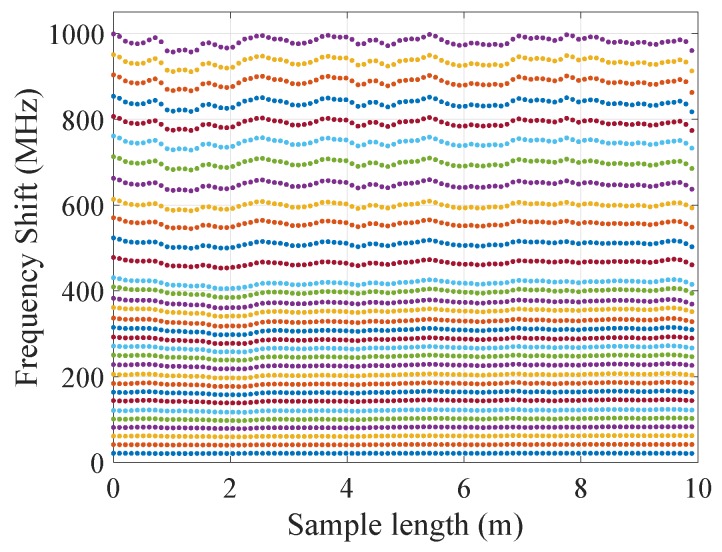
Frequency shift traces during traction (higher frequency shift for higher traction), Sample a, showing no relaxation of the sample.

It is remarkable how the cabled samples (V9 and FIMT type) show a permanent frequency shift, i.e., residual strain, after being elongated. This does not happen for the fiber, which undergoes only a slight relaxation (Figure 9). This is related to the multilayered nature of the cable: parts of it underwent permanent strain, and there may have also been slippage at the interface between the layers. The steel layer of the two cable types samples has in fact an elasto-plastic behavior and a higher Young’s modulus compared to the polyamide layer, whose behavior is visco-elastic. For this reason, steel led the mechanical behavior of the whole cable, limiting the relaxation effect of the PA, especially when it reached its plastic zone (imposed strain >2000 με). Moreover, looking at raw measurements (Figure 10), we do not observe any relaxation caused by the imposed strain; otherwise, a significant and negative slope while increasing strain should have been observed. Furthermore, as each strain level was maintained for about five minutes, a consistent noise would be visible.

This underlines the importance of characterizing the whole sensing cable and not only the fiber in primary coating for the sensitivity.

This is similar whatever the fiber (custom radiation hard or standard), as the main actors in this behavior are the protective layers of the fiber (polyamide, steel tube) and whether the samples were irradiated or not. From about 2000 με, the samples were increasingly and permanently deformed, reaching about 60 MHz for Brillouin and −170 GHz for Rayleigh scatterings, which correspond to about 1400 με, a non-negligible value (if the strain range reaches 10,000 με, plasticity is an important phenomenon to consider in the design phase). For the Cigéo reference scenario (the mechanical monitoring of radioactive waste repository cells), as the foreseen maximum strain would be around ±3000 με, the error due to this permanent strain is practically none (around 100 με in compression).

**Figure 11:**
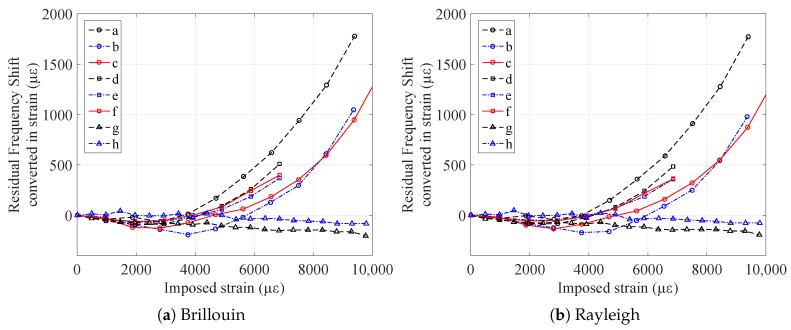
Residual strain of the samples after elongation (represented in the *x* axis) of all tested samples.

Using the strain sensitivity coefficients previously calculated, it was possible to check whether the sensors, interrogated with two different scatterings, measured the same strain values. Using the values in Table 2, it is possible to transform the frequency shift into strain following (1). The results are plotted in Figure 11.

The two strain profiles, deriving from Brillouin and Rayleigh scatterings, are çery close to each other. The results are in general in agreement with the conclusions drawn from Figure 7: FIMT type samples (*d*, *e*, and *f*) are the most plasticized, with a higher residual strain with respect to samples *b* and *c* (the custom V9, not irradiated, and irradiated). However, sample *a* is the one that shows the highest permanent strain between all, even if it is of the V9 type. This behavior, which has yet to be explained, could be due to the different adhesion between the fiber and the internal surface of the FIMT. In any case, as for the sensitivity, there is practically no difference between not irradiated and irradiated samples (*b* and *c*, *e* and *f*), considering as negligible the impact of the absorbed dose. This is very important for an environment where radiation is present, as this means that the behavior of the sensor is practically not impacted by it, then being suitable to work in such applications.

Even if Cigéo repository cells are not concerned with the plasticity of the cable during the monitoring phase, it is very important to keep in mind that its conditions may change with an unexpected rise in the strain. Nevertheless, this is a general useful reminder for all kinds of applications where the strain is over 4000 με.

The difference between the residual strains obtained with Rayleigh and Brillouin scattering is lower than the uncertainty on the Brillouin measurements (of the order of 20 με) up to 6000 με of imposed strain and remains smaller than 10% in relative value for higher imposed strains. This is very positive, as it shows that the results are the same despite the use of two interrogation methods based on two different scatterings, underlining the interoperability of the two. Moreover, since the measurement principles were different, it proves that the residual strain is related only to the modification of the cable’s structure (i.e., not on the backscattering properties).

At this point, (i) strain results acquired from the tested cables, i.e., the strain sensitivities obtained fitting the results in the imposed strain zones <2000 με and >2000 με, (ii) the independence of the cable’s behavior from radiations influence, as well as (iii) the results regarding the residual strain showed that the considered cable and its components could be operable for long term measurement. In fact, if the history of the stresses suffered by the cable were known, it would be possible to discriminate the residual strains of the cable from the elastic strains underwent at a given moment. In addition, the work done on the calibration of the strain sensitivity coefficients allowed to choose the most appropriate frequency-strain conversion coefficient for the actual state of the optical cable at the time of measurement. Thus, it would still be possible to perform strain measurements on structures, even at large strain levels, with however a slightly degraded accuracy related to the loss of linearity of the sensor. This opens up the perspective of using the instrumentation deployed, for example in Cigéo, well beyond the conditions for which the system was originally designed.

## 7. Conclusions

This paper assessed the mechanical characteristics of an optical fiber strain sensing cable, composed of layers of steel and polyamide external sheath. The considered samples, i.e., the cable at its whole, the steel tube alone, and the optical fiber in primary coating, were tested in order to analyze their strain sensitivity and the elasto-plastic behavior. These two topics were examined under two aspects: (i) the influence of the different layers of the cable and (ii) the impact of radiation on the mechanical behavior on the samples. In fact, the protective layers that composed the cable influenced both the strain sensitivity and the elasto-plastic behavior. The strain sensitivity changed at most 12% going from the one of the bare fiber (external primary coating only) to the complete cable (fiber, steel tube, and PA external sheath), while the cable itself was more plasticized than the fiber due to the presence of the steel tube, which was more ductile than glass. Radiation impact was not significant, at least for the tested total dose of 500 kGy: at most, the change in strain sensitivity coefficient was 4% (between irradiated and not irradiated samples), which is a very promising result for monitoring nuclear structure. The same was observed for the elasto-plastic behavior, which was practically unchanged in accelerated aging conditions whether the sample absorbed a radiation dose or not. The presence of other materials than the glass allowed the sensor to show a plastic behavior after 0.2% of strain (2000 με), which is the elasto-plastic limit for steel. After reaching 10,000 με of imposed strain, the cabled samples underwent from 1300 to 2000 με of residual strain even if the sample was relaxed and not elongated, which is not negligible for some applications. For our case, as the maximum strain to be reached is around ±3000 με, this paper confirmed the feasibility to use this kind of optical fiber strain sensing cable. In the future, a similar analysis is to be done for the thermal sensitivity of strain sensing cables, while a deeper investigation on the origins of the residual strain of the samples and on other mechanical behaviors (as under curvature) might be planned.

## Figures and Tables

**Table 1:** Tested samples.

	V9	FIMT	Fiber
Standard SMF G657 acrylate coating	*a*	*d*	*g*
Custom (not irradiated) SMF F doped carbon acrylate coating	*b*	*e*	*h*
Custom (irradiated) SMF F doped carbon acrylate coating	*c*	*f*	-

**Table 2:** Strain sensitivity coefficients of the different tested samples.

B: MHz/με R: GHz/με	Custom				Standard	
	Not irradiated		Irradiated			
	<0.2%	>0.2%	<0.2%	>0.2%	<0.2%	>0.2%
V9	B: 0.045	B: 0.046	B: 0.040	B: 0.045	B: 0.042	B: 0.045
	R: −0.13	R: −0.14	R: −0.12	R: −0.13	R: −0.14	R: −0.14
FIMT	B: 0.041	B: 0.047	B: 0.040	B: 0.047	B: 0.037	B: 0.045
	R: −0.12	R: −0.14	R: −0.12	R: −0.14	R: −0.11	R: −0.14
Fiber	B: 0.050	B: 0.052	B: -	B: -	B: 0.046	B: 0.049
	R: −0.15	R: −0.15	R: −	R: −	R: −0.15	R: −0.15
